# SARS-CoV-2 transmission: a sociological review

**DOI:** 10.1017/S095026882000240X

**Published:** 2020-10-07

**Authors:** Emily J. Siff, Ghazal Aghagoli, Benjamin Gallo Marin, Elizabeth Tobin-Tyler, Patricia Poitevien

**Affiliations:** 1 Biological & Biomedical Sciences, Yale University, New Haven, Connecticut, USA; 2 Warren Alpert Medical School of Brown University, Providence, Rhode Island, USA

**Keywords:** Coronavirus, COVID-19, SARS-CoV-2, sociology, virus

## Abstract

The current pandemic is defined by the transmission of severe acute respiratory syndrome coronavirus 2 (SARS-CoV-2), the virus that can lead to coronavirus disease 2019 (COVID-19). How is SARS-CoV-2 transmitted? In this review, we use a global lens to examine the sociological contexts that are potentially and systematically involved in high rates of SARS-CoV-2 transmission, including lack of personal protective equipment, population density and confinement. Altogether, this review provides an in-depth conspectus of the current literature regarding how SARS-CoV-2 disproportionately impacts many minority communities. By contextualising and disambiguating transmission risks that are particularly prominent for disadvantaged populations, this review can assist public health efforts throughout and beyond the COVID-19 pandemic.

## Introduction

While there are hundreds of coronaviruses, only seven are known to infect humans. Four of these human coronavirus strains generally have mild consequences. In sharp contrast, the other three human coronavirus strains can have severe consequences (SARS-CoV, Middle-East respiratory syndrome (MERS-CoV), SARS-CoV-2). As of August 2020, of the three most severe strains, only one has caused a pandemic: severe acute respiratory syndrome coronavirus 2 (SARS-CoV-2), the virus that can lead to coronavirus disease 2019 (COVID-19) [1].

A pandemic is defined by a disease's international or global spread [2]. Hence, pinpointing recurring disease transmission mechanisms, environments and impacts is critical for public health. Accordingly, in this review, we ask: which sociological contexts are crucially involved in the rapid and global transmission of SARS-CoV-2? To the best of our knowledge, this is the first review to provide an in-depth, global examination of the sociological scales that delineate SARS-CoV-2 transmission.

## Overview: the sociology of SARS-CoV-2 transmission

The world is a different place than it was during the 2002–2004 SARS-CoV epidemic. Throughout the 21st century, research has consistently warned that both globalisation and our exponentially increasing population could lead to higher risks of pandemics [3]. Enclosed spaces, greater population density, more travel and, consequently, more frequent human-to-human interactions all define sociological contexts that can facilitate the spread of a disease.

In this review, we delve into global spaces in order to emphasise the recurrent relationship between high-risk sociological contexts and minority communities. This review is by no means an exhaustive overview of all global settings. The objective of this review is to use a subset of international data to highlight how recurrent and disproportionately impactful sociological contexts are tied to high rates of SARS-CoV-2 transmission.

## In-depth: disproportionate impacts on minority communities

Across the globe, minority communities are experiencing a disproportionately high risk of COVID-19 infection, hospitalisation and death. These international patterns possess a straightforward homogeneity: from the United States (US) to the United Kingdom (UK) to Brazil, the syndemic, synergistic relationship between the COVID-19 pandemic and historically institutionalised biases and racism has resulted in a globally disproportionate lethality [4–6].

### High infection and hospitalisation rates

Spanning across the United Kingdom (UK), Brazil, Norway, Sweden and the United States (US), international data consistently suggest that minority communities are subject to disproportionately high rates of infection and hospitalisation. Although a recent UK census indicates that racial and ethnic minorities comprise about 14% of the UK population, on 1 May 2020, the UK's Intensive Care National Audit and Research Centre reported that racial and ethnic minorities made up 2300 (34%) of 6770 critically ill COVID-19 patients [7].

Analogously, using multivariate logistic regression, clinical data from Brazil revealed that, of 10 713 COVID-19 positive patients, only those who had Asian, indigenous or ‘unknown race’ backgrounds were at increased risk for hospitalisation [8]. In Norway, individuals with Somalian backgrounds have had COVID-19 infection rates that are greater than 10 times the national average, representing 1586 per 100 000 (*vs.* national average: 140 per 100 000). In Sweden, a Public Health Agency survey demonstrated that, despite comprising 0.5% of Sweden's population, immigrants from Somalia, Syria and Iraq made up 5% of confirmed COVID-19 cases [9]. Broadly, throughout Europe, the Roma minority has experienced staggeringly disproportionate COVID-19 risks [5].

Moreover, a recent US study that analysed data from 14 states found that, of 580 COVID-19 positive hospitalised patients, 261 (45.0%) were non-Hispanic white (‘white’), 192 (33.1%) were non-Hispanic Black (‘Black’), 47 (8.1%) were Hispanic, 32 (5.5%) were Asian, 2 (0.3%) were American Indian/Alaskan Native and 46 (7.9%) were of other or unknown race [10]. Given that, for example, approximately 76.5% of the US population is white while only 13.4% of the US population is Black (United States Census Bureau, 2019) [11], these COVID-19 hospitalisation rates of 45.0% white *vs.* 33.1% Black are disproportionate. Finally, in terms of indigenous communities, the Navajo Nation in the US surpassed all of New York in terms of numbers of per capita COVID-19 cases [12].

### High death rates

On an international scale, COVID-19 deaths are recurrently and disproportionately impacting minority communities. In the UK, even when controlling for differences in frontline worker positions, data indicate that healthcare staff in the National Health Service (NHS) who identify as racial and ethnic minorities have experienced disproportionate COVID-19 mortality rates [7]. Furthermore, an analysis run the UK Office for National Statistics has indicated that, compared to white individuals in the UK, risk of COVID-19-related death is much higher for many racial and ethnic minorities in the UK. In particular, after controlling for age, the analysis revealed that, compared to white men and white women, Black men and Black women are, respectively, 4.2 and 4.3 more likely to experience COVID-19-related deaths while UK individuals of Bangladeshi, Pakistani, Indian or mixed race backgrounds had a statistically significant higher risk of COVID-19-related mortality [13]. Consistently, another study found that, while Black individuals in the UK were more than four times more likely to die from COVID-19, individuals in the UK of Bangladeshi, Pakistani, Chinese and mixed race backgrounds were approximately 1.8 times more likely to experience COVID-19-related mortality [9].

In Brazil, one report suggested that its indigenous communities are potentially experiencing double the amount of COVID-19 deaths than the rest of its population [12]. Additionally, of 11 321 confirmed COVID-19 positive hospitalised patients in Brazil, mixed race (*Pardo*) and Black (*Preto*) patients had a significantly higher risk of mortality than white (*Branco*) patients (hazard ratio, 95% CI 1.45–1.33–1.58 for *Pardo*; 1.32, 1.15–1.52 for *Preto*). Moreover, after age, mixed race was the second most meaningful risk factor in predicting death [14]. In the US, a set of New York City data revealed that death rates for Black individuals (92.3 deaths per 100 000) and Hispanic individuals (74.3 deaths per 100 000) were higher than death rates for white individuals (45.2 deaths per 100 000) and Asian individuals (34.5 deaths per 100 000; Bureau of Communicable Disease Surveillance System, 2020) [15].

## In-depth: sociological contexts in minority communities

Disproportionate impacts on minority communities are incontrovertibly tied to disproportionate exposure to high-risk transmission contexts. Broadly, we define contexts linked to higher SARS-CoV-2 transmission as enclosed environments that (a) lack critical resources, including personal protective equipment (PPE), (b) have a high population density, (c) and have low population mobility.

### At work

Transmission risks are particularly high for individuals in industries designated as essential, in that they must continue to work on the frontlines despite outbreaks. In particular, high rates of transmission and death have occurred in the service industry and factory-farming industry, including meatpacking facilities [16]. These contexts are generally comprised of minimal resources (such as protective equipment), densely packed working conditions, low wages and low mobility. Low mobility includes the inability to leave or take time off from a dangerous job due to financial pressure. In short, individuals in low-wage occupations are often working in enclosed, high-transmission contexts without the financial option to leave.

To a skewed degree, many of the frontline workers in these essential industries are people of color and immigrants. For instance, in the US, low-wage, frontline occupations are disproportionately comprised of Black individuals, Hispanic individuals and immigrants [4, 17]. Specifically, Hispanic individuals account for 17% of the total US workforce; yet approximately 53% of individuals working in agricultural sectors are Hispanic. Analogously, Black individuals comprise about 12% of the total US workforce; yet approximately 36% of individuals working as nursing, psychiatric or health home aides, 31% of individuals working as security guards and surveillance gaming officers and 30% of individuals working as licensed practical and licensed vocational nurses are Black [18]. Compounding transmission inequities, individuals working in low-wage sectors may be unable to leave their occupations or receive healthcare due to lack of living wages, no paid sick leave and/or no health insurance, which people of color in the US disproportionately lack access to [4, 19]. Without access to these essential healthcare resources, individuals may be unable to take time off from work, despite being a carrier for SARS-CoV-2.

Undeniably, many essential industries are high-risk transmission environments [4, 17]. For instance, from 9 March to 20 April 2020, a total of 2494 COVID-19 deaths from the working age population (aged 20–64) of the UK were recorded. Compared to the death rates among people of the same sex and age in the general population, the following groups had significantly higher COVID-19 death rates: (a) males working as security guards (45.7 deaths per 100 000), (b) males working in construction (25.9 deaths per 100 000), (c) males working in service occupations (19.3 deaths per 100 000 males) and (d) males and females working in social care (which includes transport workers; 23.4 deaths per 100 000) [20]. Analogously, a recent Centers for Disease Control and Prevention (CDC) study found that, in just 239 US meat and poultry processing facilities, 16 233 COVID-19 positive cases were reported throughout April and May. Among the 9919 cases in which race and ethnicity were recorded, racial and ethnic minorities comprised 87% of COVID-19 positive individuals [16].

### At home

Racial and ethnic minorities disproportionately live in densely populated areas, which can contribute to high transmission rates [21]. Specifically, racial residential segregation is tied to higher population density and greater health disparities, such as a greater distance to facilities, a larger frequency of adverse health outcomes, including mortality [22] and a higher likelihood of underlying health conditions, including diabetes [23]. In brief, with more comorbidities and less access to necessary facilities, such as grocery stores and hospitals, many people of color, immigrants and indigenous communities are particularly susceptible to SARS-CoV-2 transmission and mortality.

### In health

In the UK, compared to white people, disproportionate risks of in-hospital COVID-19 deaths for Black individuals and individuals with Asian backgrounds have been consistently attributed to deprivation [9, 24]. In Brazil, that white (Branco) individuals are more likely to survive COVID-19 than mixed race (Pardo) individuals has been partially attributed to data that reveal (a) white individuals are more likely to be admitted to the ICU once hospitalised and (b) white individuals have greater access to private hospital settings [14]. In the US, multivariate regression modelling from four distinct datasets consistently indicated that, compared to white individuals, Hispanic individuals have statistically significantly lower rates of paid-leave access [19]. Moreover, across ages, Black individuals were found to be far more likely than white individuals to report not having been able to see a doctor in the past year due to cost, regardless of desire or need [25]. Finally, the median household income for Native Americans on or affiliated with US tribal reservations is approximately $39 700 (a sharp contrast to the median household income for the rest of the US, $57 600) and one of the primary income sources, casinos, are now completely shut down. With Native American tribes' tax base drastically cut, tribal governments no longer have the funds to run health clinics, significantly undermining access to healthcare [26].

Racial bias can also occur in the healthcare system, which (a) may lead to decreased trust in healthcare and (b) has been strongly tied to worse treatment outcomes for people of color. For instance, a pivotal study that included white medical students and white residents demonstrated that some falsely believed there were biological differences in Black and white individuals; these false beliefs predicted a racial bias in pain perception and treatment recommendation accuracy [27]. In terms of COVID-19, data have already begun to suggest that, when reporting the same symptoms as white individuals, Black and Hispanic individuals are less likely to receive testing and treatment [28].

Finally, a key question is: how feasible is access to medical care for detainees? A recent US study used a stochastic susceptible−exposed−infected−recovered model to (a) estimate the rate of SARS-CoV-2 transmission within 111 US immigration and Customs Enforcement (ICE) detention centres and (b) evaluate the impacts on regional hospital intensive care unit (ICU) capacity. The study found that, assuming the most optimistic parameters (*R*_0_ = 2.5), 72% of individuals would be expected to be infected by day 90. With more pessimistic parameters (*R*_0_ = 7), nearly 100% of individuals would be expected to be infected by day 90. Assuming all ICU beds were made available for infected detainees, in the most optimistic scenario, SARS-CoV-2 outbreaks among a minimum of 66 ICE facilities (59%) would overwhelm ICU beds within a 10-mile radius over a 90-day period; outbreaks among a minimum of nine ICE facilities (8%) would overwhelm local ICU beds within a 50-mile radius over a 90-day period [29]. Hence, the impact of SARS-CoV-2 transmission in detention facilities is both lethal within and beyond these spaces.

### In public spaces

Ranging from highly efficient filtration respirators to less efficient cloths, a critical component of reducing transmission is wearing a mask. Even cloth can help the wearer reduce viral transmission (if infected) and, conversely, can protect one from becoming infected [30]. However, reports have indicated that Black individuals, particularly Black men, may not wear protective masks because they are concerned about experiencing greater racial profiling from police [31]. Racial profiling is characterised as consciously or subconsciously using an individual's race or ethnicity as the basis for suspecting them of having committed an offense. Across the globe, from the UK to Canada to India to South Africa to Japan to the US, people of color and immigrants have historically been disproportionately subjected to police contact, scrutiny and force, which has frequently been attributed to racial profiling [32–34]. In brief, the stereotypes, bias and racism that many people of color and immigrants already face may be an additional barrier to safely protecting themselves and others from SARS-CoV-2 transmission.

### In incarceration

As enclosed spaces with high population densities, minimal resources, and few mobility options, incarcerated spaces are breeding grounds for viral transmission. As of 15 March 2020, the World Health Organization (WHO) issued suggestions for prisons and other places of detention to implement [35]. While the suggestions to protect the health, safety and human rights of incarcerated individuals are sound, many facilities cannot or will not implement this guidance due to a combination of being understaffed, lacking funding for and access to basic medical supplies and prioritising security over health [36]. Due to institutionalised racism, communities of color are often disproportionately represented in incarcerated spaces [37] and, therefore, will disproportionately bear the negative impacts of SARS-CoV-2 transmission.

Occurring on massive, rapid and global scales, many prison and jail outbreaks and deaths from COVID-19 have occurred. For instance, in Wuhan, China, almost half of reported COVID-19 incident cases (233 of 565) were from the prison system. Analogously, in Shendong, China, 450 miles away, a separate, major prison outbreak was traced back to officials who had visited Wuhan and then infected seven prison guards and 200 inmates [38]. Similarly, in the US, a state prison in Ohio reported that 78% of inmates (2011 individuals) tested positive for COVID-19. As of 22 April 2020, Ohio's incarcerated population makes up 20% of the entire state's positive coronavirus cases [39]. In Brazil, an estimated 80% of the prison population will be infected with COVID-19, of which 20% may need hospitalisation [40]. Importantly, although inmates are confined, jail and prison guards are also exposed to SARS-CoV-2; on a daily basis, guards actively exit the complexes. Therefore, jail and prison outbreaks are not isolated events; SARS-CoV-2 outbreaks in these confined spaces may easily result in major transmission events in surrounding areas.

Notably, prisons, jails and ICE detention centres may be privately owned. When private ownership applies, these spaces are not subject to crucial governmental jurisdictions. Private companies manage 8.4% of the US prison population and 5.4% of the US jail population [41]. Privately owned prisons and jails have received attention for human rights violations due to overcrowding, an issue that is particularly deadly during a pandemic. Most detained US immigrants are housed in one of the 250+ local detention facilities that operate under either intergovernmental service agreements or private facilities. These facilities are not subject to the Performance-Based National Detention Standards [42].

## Summary

Although SARS-CoV-2 transmission is complex, there are common sociological contexts that contribute to transmission rates, such as lack of access to PPE, population density and confinement. Delving into data on a global scale, this review has emphasised how minority communities' disproportionate exposure to high-risk transmission contexts is often closely tied to disproportionate rates of transmission, hospitalisation and death. The current pandemic has scathingly underscored that biology and sociology are inextricably linked: a consideration of sociological conditions is necessary when addressing and mitigating the noxious and unequal consequences of viral transmission. By contextualising and disambiguating the transmission risks that disadvantaged communities uniquely face, our review can help guide public health efforts throughout and beyond the COVID-19 pandemic.

## Data Availability

The findings in this review do not rely upon any data, code or other resources personally generated by EJS, GA, BGM, ETT or PP. The information in this review is derived from pre-existing literature, all of which is listed in the references and available online.

## References

[1] Lu R (2020) Genomic characterisation and epidemiology of 2019 novel coronavirus: implications for virus origins and receptor binding. Lancet 395, 565–574. 32007145 10.1016/S0140-6736(20)30251-8 PMC7159086

[2] Heath K (2011) The classical definition of a pandemic is not elusive. Bulletin of the World Health Organization 89, 540–541. 21734771 10.2471/BLT.11.088815 PMC3127276

[3] Institute of Medicine (U.S.) Forum on Microbial Threats (2006) Workshop Summary. Washington, DC: National Academies Press, pp. 1–228. doi:10.17226/11588.

[4] Poteat T (2020) Understanding COVID-19 risks and vulnerabilities among black communities in America: the lethal force of syndemics. Annals of Epidemiology 47, 1–3. 32419765 10.1016/j.annepidem.2020.05.004 PMC7224650

[5] Matache M and Bhabha J (2020) Anti-Roma racism is spiraling during COVID-19 pandemic. Health and Human Rights 22, 379–382, PMCID: PMC7348427. 32669824 PMC7348427

[6] Barber S (2020) Death by racism. The Lancet Infectious Diseases 20, 903. 32738238 10.1016/S1473-3099(20)30567-3

[7] Intensive Care National Audit and Research Centre (2020) INARC report on COVID-19 in critical care. INARC. Available at https://www.icnarc.org/OurAudit/Audits/Cmp/Reports (Accessed 5 August 2020).

[8] Soares RCM, Mattos LR and Raposo LM (2020) Risk factors for hospitalization and mortality due to COVID-19 in Espírito Santo State, Brazil [published online ahead of print, 2020 July 16]. The American Journal of Tropical Medicine and Hygiene **103**(3), 1184–1190. doi: 10.4269/ajtmh.20-0483. PMC7470570 32682453

[9] Yaya S (2020) Ethnic and racial disparities in COVID-19-related deaths: counting the trees, hiding the forest. BMJ Global Health 5, e002913. 10.1136/bmjgh-2020-002913 PMC7298686 32513864

[10] Garg S (2020) Hospitalization rates and characteristics of patients hospitalized with laboratory-confirmed coronavirus disease 2019 – COVID-NET, 14 States, March 1–30. Morbidity and Mortal Weekly Report (MMWR) 69, 458–464. 10.15585/mmwr.mm6915e3 PMC7755063 32298251

[11] United States Census Bureau. U.S. Census Bureau, Population Estimates Program (PEP) (2019) Population and Housing Unit Estimates 2019 U.S. Census Bureau, American Community Survey (ACS); American Community Survey. Available at https://www.census.gov/quickfacts/fact/table/U.S./RHI225218#RHI225218 (Accessed 20 May 2020).

[12] Curtice K and Choo E (2020) Indigenous populations: left behind in the COVID-19 response. The Lancet 395, 1753. 10.1016/S0140-6736(20)31242-3 PMC7272170 32505246

[13] Office for National Statistics (2020) Coronavirus (COVID-19) related deaths by ethnic group, England and Wales. *Office for National Statistics* Available at https://www.ons.gov.uk/peoplepopulationandcommunity/birthsdeathsandmarriages/deaths/articles/coronavirusrelateddeathsbyethnicgroupenglandandwales/2march2020to10april2020#ethnic-breakdown-of-deaths-by-age-and-sex (Accessed 20 May 2020).

[14] Baqui P (2020) Ethnic and regional variations in hospital mortality from COVID-19 in Brazil: a cross-sectional observational study. The Lancet Global Health 8, e1018–e1026. 32622400 10.1016/S2214-109X(20)30285-0 PMC7332269

[15] Bureau of Communicable Disease Surveillance System (2020) Age-adjusted rates of lab confirmed COVID-19 non hospitalized cases, estimated non-fatal hospitalized cases, and patients known to have died 100 000 by race/ethnicity group as of 16 April 2020. *New York City* (*NYC*) *Health* Available at https://www1.nyc.gov/assets/doh/downloads/pdf/imm/covid-19-deaths-race-ethnicity-04162020-1.pdf (Accessed 20 May 2020).

[16] Waltenburg MA (2020) Update: cOVID-19 among workers in meat and poultry processing facilities — United States, April–May 2020. Morbidity and Mortal Weekly Report (MMWR) 69, 887–892. 10.15585/mmwr.mm6927e2 PMC7732361 32644986

[17] Clark E (2020) Disproportionate impact of the COVID-19 pandemic on immigrant communities in the United States. PLoS Neglected Tropical Diseases 14, e0008484. 32658925 10.1371/journal.pntd.0008484 PMC7357736

[18] U.S. Bureau of Labor Statistics (2018) Report 1082, Labor force characteristics by race and ethnicity, Bureau of Labor Statistics (BLS) Reports 2019. Available at https://www.bls.gov/opub/reports/race-and-ethnicity/2018/home.htm (Accessed 19 May 2020).

[19] Bartel AP (2019) Racial and ethnic disparities in access to and use of paid family and medical leave: evidence from four nationally representative datasets. Monthly Labor Review, U.S. Bureau of Labor Statistics. doi: 10.21916/mlr.2019.2.

[20] Windsor-Shellard B and Kaur J (2020) Coronavirus (COVID-19) related deaths by occupation, England and Wales: deaths registered up to and including 20 April 2020. Provisional analysis of deaths involving the coronavirus (COVID-19), by different occupational groups, among males and females aged 20 to 64 years in England and Wales. Office for National Statistics. Available at https://www.ons.gov.uk/peoplepopulationandcommunity/healthandsocialcare/causesofdeath/bulletins/coronaviruscovid19relateddeathsbyoccupationenglandandwales/deathsregistereduptoandincluding20april2020 (Accessed 11 May 2020).

[21] Rocklöv J and Sjödin H (2020) High population densities catalyse the spread of COVID-19. Journal of Travel Medicine 27, taaa038. 32227186 10.1093/jtm/taaa038 PMC7184409

[22] Hearst MO, Oakes JM and Johnson PJ (2008) The effect of racial residential segregation on black infant mortality. American Journal of Epidemiology 168, 1247–54. 18974059 10.1093/aje/kwn291

[23] Bravo MA (2018) Residential racial isolation and spatial patterning of type 2 diabetes mellitus in Durham, North Carolina. American Journal of Epidemiology 187, 1467–1476. 29762649 10.1093/aje/kwy026

[24] Williamson E (2020) OpenSAFELY: factors associated with COVID-19-related hospital death in the linked electronic health records of 17 million adult NHS patients. Nature 588, 430–436. 10.1038/s41586-020-2521-4 PMC7611074 32640463

[25] Cunningham TJ (2017) Vital signs: racial disparities in age-specific mortality among blacks or African Americans – United States, 1999–2015. Morbidity and Mortal Weekly Report (MMWR) 66, 444. 10.15585/mmwr.mm6617e1 PMC5687082 28472021

[26] Dorn AV, Cooney RE and Sabin ML (2020) COVID-19 exacerbating inequalities in the US. The Lancet 395, 1243–1244. 10.1016/S0140-6736(20)30893-X PMC7162639 32305087

[27] Hoffman KM (2016) Racial bias in pain assessment and treatment recommendations, and false beliefs about biological differences between blacks and whites. Proceedings of the National Academy of Sciences of the United States of America 113, 4296–4301. 27044069 10.1073/pnas.1516047113 PMC4843483

[28] Rubix, Life Sciences (2020) COVID-19 & Minority Health Access. Discovery Report. Available at https://rubixls.com/2020/04/01/health-data-in-the-covid-19-crisis-how-racial-equity-is-widening-for-patients-to-gain-access-to-treatment/ (Accessed 20 May 2020).

[29] Irvine M (2020) Modeling COVID-19 and impacts on U.S. Immigration and Enforcement (ICE) detention facilities. Journal of Urban Health **97**. doi: 10.1007/s11524-020-00441-x. PMC7228433 32415422

[30] Feng S (2020) Rational use of face masks in the COVID-19 pandemic. The Lancet Respiratory Medicine 8, 434–436. 32203710 10.1016/S2213-2600(20)30134-X PMC7118603

[31] Taylor DB (2020) For Black Men, Fear That Masks Will Invite Racial Profiling. *New York Times* Available at https://www.nytimes.com/2020/04/14/us/coronavirus-masks-racism-african-americans.html (Accessed 20 May 2020).

[32] Weber L and Bowling B (2014) Stop and Search: Police Power in Global Context. Milton Park, Abingdon, Oxon: Routledge.

[33] Milner AN, George BJ and Allison DB (2016) Black and Hispanic men perceived to be large are at increased risk for police frisk, search, and force. PLoS ONE 11, e0147158. 26785118 10.1371/journal.pone.0147158 PMC4718646

[34] Pierson E (2020) A large-scale analysis of racial disparities in police stops across the United States. Nature Human Behaviour 4, 736–745. 10.1038/s41562-020-0858-1 32367028

[35] World Health Organization (WHO) (2020) Preparedness, prevention and control of COVID-19 in prisons and other places of detention, Interim guidance, 15 March 2020. *Regional Office for Europe* Available at http://www.euro.who.int/__data/assets/pdf_file/0019/434026/Preparedness-prevention-and-control-of-COVID-19-in-prisons.pdf?ua=1 (Accessed 20 May 2020).

[36] Williams B (2020) Correctional facilities in the shadow of COVID-19: unique challenges and proposed solutions. Health Affairs. doi: 10.1377/hblog20200324.784502.

[37] Alexander M (2012) The New Jim Crow: Mass Incarceration in the Age of Color Blindness. *The New Press* ISBN-13: 978-1595586438.

[38] Barnert E, Ahalt C and Williams B (2020) Prisons: amplifiers of the COVID-19 pandemic hiding in plain sight. American Journal of Public Health 110, 964–966. doi: 10.2105/AJPH.2020.305713. 32407126 PMC7287517

[39] Bates J (2020) Ohio Began Mass Testing Incarcerated People for COVID-19. The Results Paint a Bleak Picture for the U.S. Prison System. *TIME* 2020. Available at https://time.com/5825030/ohio-mass-testing-prisons-coronavirus-outbreaks/ (Accessed 20 May 2020).

[40] Shadmi E (2020) Health equity and COVID-19: global perspectives. International Journal for Equity in Health 19, 104. Published 2020 June 26. 32586388 10.1186/s12939-020-01218-z PMC7316580

[41] Gaes GG (2019) Current status of prison privatization research on American prisons and jails. Criminology & Public Policy 18, 269–293.

[42] Page K (2020) Undocumented U.S. Immigrants and Covid-19. New England Journal of Medicine 382, e62. 32220207 10.1056/NEJMp2005953

